# *Lactobacillus rhamnosus* GG and *Bifidobacterium animalis* subsp. *lactis* BB‐12 promote infected wound healing via regulation of the wound microenvironment

**DOI:** 10.1111/1751-7915.70031

**Published:** 2024-10-18

**Authors:** Zhe Yin, Yilin Wang, Xiaojuan Feng, Changqing Liu, Xiaoyang Guan, Shuyan Liu, Zhanyi Long, Zhonghua Miao, Fang He, Ruyue Cheng, Yanting Han, Ka Li

**Affiliations:** ^1^ Sichuan University—The Hong Kong Polytechnic University Institute for Disaster Management and Reconstruction Chengdu China; ^2^ Medicine and Engineering Interdisciplinary Research Laboratory of Nursing & Materials, West China Hospital, Sichuan University/West China School of Nursing Sichuan University Chengdu China; ^3^ Department of Gastroenterology Affiliated Tumor Hospital of Xinjiang Medical University Urumqi China; ^4^ Department of General Surgery West China Hospital, Sichuan University Chengdu China; ^5^ School of Fashion and Textiles The Hong Kong Polytechnic University Kowloon Hong Kong SAR China; ^6^ Department of Clinical Nutrition, West China Second Hospital Sichuan University Chengdu China; ^7^ Department of Nutrition and Food Hygiene, West China School of Public Health and West China Fourth Hospital Sichuan University Chengdu China

## Abstract

Infected wounds can result in complex clinical complications and delayed healing, presenting a significant global public health challenge. This study explored the effects of topical application of two probiotics, *Lactobacillus rhamnosus* GG (LGG) and *Bifidobacterium animalis* subsp. *lactis* BB‐12, on the microenvironment of infected wounds and their impact on wound healing. LGG and BB‐12 were applied separately and topically on the *Staphylococcus aureus (S. aureus)*‐infected skin wounds of the rat model on a daily basis. Both probiotics significantly accelerated wound healing, demonstrated by enhanced granulation tissue formation and increased collagen deposition, with BB‐12 showing superior efficacy. LGG and BB‐12 both effectively inhibited neutrophil infiltration and decreased the expression of pro‐inflammatory cytokines tumor necrosis factor‐α (TNF‐α) and interleukin‐6 (IL‐6). Notably, BB‐12 markedly reduced IL‐6 levels, while LGG significantly lowered TNF‐α, transforming growth factor‐β (TGF‐β) and vascular endothelial growth factor (VEGF). Additionally, both probiotics promoted macrophage polarization towards the anti‐inflammatory M2 phenotype. Microbiota analysis revealed that LGG and BB‐12 significantly decreased the abundance of pathogenic bacteria (e.g. *Staphylococcus* and *Proteus*) and increased the proportion of beneficial bacteria (e.g. *Corynebacterium*). Particularly, BB‐12 was more effective in reducing *Staphylococcus* abundance, whereas LGG excelled in promoting *Corynebacterium* growth. These findings suggest the ability of LGG and BB‐12 to modulate the wound microenvironment, enhance wound healing and provide valuable insights for the management of infected wounds.

## Graphical abstract

This study explores the topical application of *Lactobacillus rhamnosus* GG (LGG) and *Bifidobacterium animalis* subsp. *lactis* BB‐12 on *Staphylococcus aureus*–infected wounds in a rat model, demonstrating accelerated healing and enhanced modulation of the wound microenvironment. BB‐12 showed superior efficacy in reducing inflammation and pathogenic bacteria, while both probiotics promoted collagen deposition, macrophage polarization and beneficial microbiota balance.

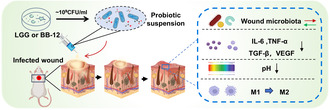

## INTRODUCTION

The wound microenvironment refers to the complex biological and biochemical environment within the wound, including cells, extracellular matrix, microbiota and wound pH (Wang, Qi, et al., 2022). Once the wound microenvironment is imbalanced, the wound is susceptible to bacterial infection, significantly hindering the healing process (Dhall et al., 2019; Mo et al., 2023). This imbalance often manifests itself as increased abundance of pathogenic microorganisms, changes in pH and impaired immune cell function, which weakens the body's defences against infection, delaying the healing processes. Therefore, regulating and restoring the balance of the wound microenvironment is essential to control wound infection (Zhang et al., 2022).

Currently, clinical treatments for infected wounds mainly include debridement, intravenous infusion, oral antibiotics and the application of antibacterial dressings (Han et al., 2024; Reigadas et al., 2021). Although these therapeutic measures aim to optimize the wound microenvironment, they are often accompanied by problems such as pain, secondary damage and bacterial resistance (Chowdhury & Findlay, 2023; Salisbury et al., 2018). Therefore, there is an increasing need to develop new safe and effective wound care technologies to promote wound healing.

Recent studies have further revealed the important role of skin microbiota in wound healing. The skin microbiota not only protects the skin from pathogens but also participates in the regulation of the immune system, thereby affecting the wound‐healing process (Constantinides et al., 2019). For example, Baquer et al. (2023) found that skin microbiota can stimulate T cells to secrete IFN‐γ while inhibiting the expression of pro‐inflammatory chemokines IL‐8, CXCL1 and MCP‐1, thereby reducing inflammatory responses. Scharschmidt et al. (2017) revealed the critical role of the skin microbiota in promoting the enrichment and localization of normal immune cells in the skin, while Enamorado et al. (2023) demonstrated that the skin microbiota can promote the repair and regeneration of sensory neurons. In addition, Wang et al. (2021) found that an imbalance in skin microbiota can inhibit the regenerative ability of skin and hair follicles. These findings indicate the critical role of the microbiota in the homeostasis of the wound microenvironment.

In this context, probiotics, as supplements to the commensal microbiota, may become a potential strategy to modulate the wound microenvironment by providing a protective barrier and modulating immune responses. Studies have attempted to explore the role of probiotics in wound healing. Ong et al. (2020) developed an ointment containing 10% protein fraction of *Lactobacillus plantarum* and applied it to wounds. The results showed that *L. plantarum* can inhibit the growth of *Staphylococcus aureus(S. aureus)*, and enhance the expression of cytokines and chemokines, thereby promoting the migration of keratinocytes and wound healing. Although topical probiotics show significant potential in wound care, results from existing studies show some heterogeneity due to differences in strain selection, dose, concentration, duration and administration method (Knackstedt et al., 2020). For example, Partlow et al. (2016) found that topical application of *Saccharomyces boulardii* did not accelerate wound healing or significantly alter the wound microbiota. Nevertheless, the diversity of these studies reflects the breadth of exploration in probiotic applications rather than methodological shortcomings.

To further advance this field, more targeted and systematic research is urgently needed, especially in clarifying the mechanisms of action of specific probiotic strains in specific wound environments. To address these issues, this study thoroughly investigates the specific roles of *Lactobacillus rhamnosus* GG (LGG) and *Bifidobacterium animalis* subsp. *lactis* BB‐12 in modulating the wound microenvironment during the healing of infected wounds. For the first time, this study systematically compares the effects of these two probiotics on a rat skin infection model regarding their ability to reduce the pathogen load, modulate inflammation, reshape the wound microbiota and accelerate wound healing. Through multi‐omics approaches, this study also uncovers the mechanism by which probiotics modulate the microbiota to enhance wound healing, providing a theoretical basis for developing new wound treatment strategies. The corresponding findings suggest that probiotics hold significant potential as alternatives or adjuncts for infected wound treatment, providing theoretical and experimental references for future research and clinical applications in this field.

## EXPERIMENTAL PROCEDURES

### Materials

LGG (ATCC 53103) and BB‐12 (ATCC 50675) were purchased from Chr. Hansen Holding A/S, Hørsholm, Denmark, and cultured in de Man, Rogosa and Sharpe (MRS) agar and TOS propionate agar respectively. *Staphylococcus aureus* (ATCC 6538) and *Escherichia coli* (ATCC 23815) were purchased from Shanghai Luwei Technology Co., Ltd., and cultured on nutrient agar. De Man, Rogosa and Sharpe (MRS), tomato Juice (TOS) propionate ager and nutrients  were purchased from Qingdao Hi‐Tech Industrial Park, Haibo Biotechnology Co., Ltd. (Qingdao, China).

### Preparation of live LGG suspension

**FIGURE 1:**
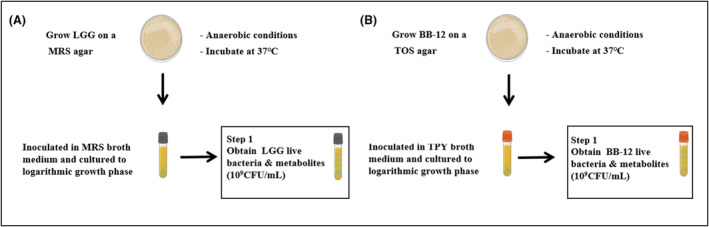
Preparation method for (A) live LGG and (B) live BB‐12 suspensions.

Live LGG was grown on MRS agar at 37°C for 48 h. From the agar, colonies were picked and inoculated in MRS broth and cultured until the logarithmic growth phase was reached. The culture was stopped when the population was approximately 10^9^ Colony‐forming unit (CFU)/mL to obtain the mixture of live LGG suspension with its metabolites. The preparation scheme is summarized in Figure 1A.

### Preparation of live BB‐12 suspension

Live BB‐12 was grown on TOS agar at 37°C for 48 h. From the agar, colonies were picked and inoculated in tryptone‐yeast (TPY) broth and cultured until the logarithmic growth phase was reached. The culture was stopped when the population was approximately 10^9^ CFU/mL to obtain the mixture of live BB‐12 suspension with its metabolites. The precreation scheme has been summarized in Figure 1B.

### In vivo experiments on infected wounds in rats

Animal experiments were approved by the Animal Ethics Committee of Sichuan University (Animal Ethics Filing Number: 20230515003) and conducted in accordance with the guidelines of the China Research Institute Animal Use Committee. Sprague Dawley (SD) male rats (8 weeks, 220 ± 10 g) were purchased from Chongqing Enswell Biotechnology Co., Ltd. Anaesthesia was induced using isoflurane gas at an induction concentration of 3%–4% and a maintenance concentration of 2%–2.5%. As described earlier (Lu et al., 2022; Yang et al., 2023), a full‐thickness skin defect wound with a diameter of 10 mm was established on the back of rats, and then 100 μL *S. aureus* bacterial suspension (10^8^ CFU/mL) was applied to the wound and covered for 24 h. The 30 rats were randomly divided into three groups: control (10 rats), LGG (10 rats) and BB‐12 (10 rats). Briefly, 500 μL of LGG or BB‐12 suspension (10^9^ CFU/mL) was applied to the wound, whereas the control group did not receive any intervention. The weight, wound pH and wound healing status of the rats were observed and recorded daily, and the wound area was measured using ImageJ software according to the following equation:

$$\text{Wound area}\;\left(\%\right)=S_{n}/S_{0}\times 100\%$$

where *S*_0_ and *S*_*n*_ are the wound areas on days 0 and n respectively. The wound area has been represented as mean ± SD.

### Blood sample analysis

Blood (0.5 mL) was collected from the tail vein of the rats before the intervention (before and after modelling) and 3 days after the intervention. Blood analysis was performed using a fully automatic veterinary blood cell analyser (Shenzhen Mindray Bio‐Medical Electronics Co. BC‐5000 Vet).

### Analysis of wound histology and immunology

Wound skin tissue was stained with H&E and Masson, and immunohistochemical studies were performed (IL‐6, TNF‐α, VEGF and TGF‐β). The primary antibody for TNF‐α was diluted 1:200 (BA0131, Boster Bio), VEGF was diluted 1:200 (bs‐1313R, Bioss), IL‐6 was diluted 1:200 (GB11117, Servicebio) and TGF‐β was diluted 1:200 (GB11117, Servicebio). Images of the stained sections were taken using the 3DHISTECH (Hungarg) CaseViewer2.4 software and the data image analysis system Indica Labs (USA) Halo 101‐WL‐HALO‐1. The wound tissue was immunofluorescent stained (CD206 and CD86) and observed under a fluorescence microscope (Eclipse E100, Nikon). The number of cells expressing CD86 and CD206 was analysed using ImageJ software.

### Analysis of wound microorganisms

Wound swab samples were collected using sterile swabs and cryovials and stored in a refrigerator set to −80°C. A Tiangen DNA kit (Tiangen Science and Technology, Beijing, China) was used to extract the DNA from the wound tissue. 16SrRNA gene fragments were amplified with universal primers (forward primer, V3‐338F5′‐ACTCCTACGGGAGGCAGCAG‐3′; reverse primer, V4‐806R 5′‐GGACTACHVGGGTWTCTAAT‐3′) (Ren et al., 2017). PCRs were conducted as 25‐μL reactions comprising 12.5 μL of the Phusion® Hot Start Flex 2X Master Mix (New England Biolabs Inc., Ipswich, MA, USA), 2.5 μL of the forward and reverse primers and approximately 50 ng of the template DNA (Cheng et al., 2018).

### Statistical analyses

Statistical analyses were performed using Origin software. All data were presented as the mean ± SD. One‐way analysis of variance (ANOVA) was used to determine significant differences between means at a significance level of *p* < 0.05. Tukey's test was used for multiple comparisons between the means. *p*‐values were calculated to indicate the significance levels, presented as *p < 0.05, **p < 0.01 and ***p < 0.001.

All experimental data were based on samples from 10 rats per group. For histological and immunofluorescence staining results, the average values from three different sections per sample were used for statistical analysis. The 16S rRNA‐sequencing data were analysed using multiple comparison tests to assess differences in microbial communities between the experimental groups.

## RESULTS

### Effect of LGG and BB‐12 on wound healing

**FIGURE 2:**
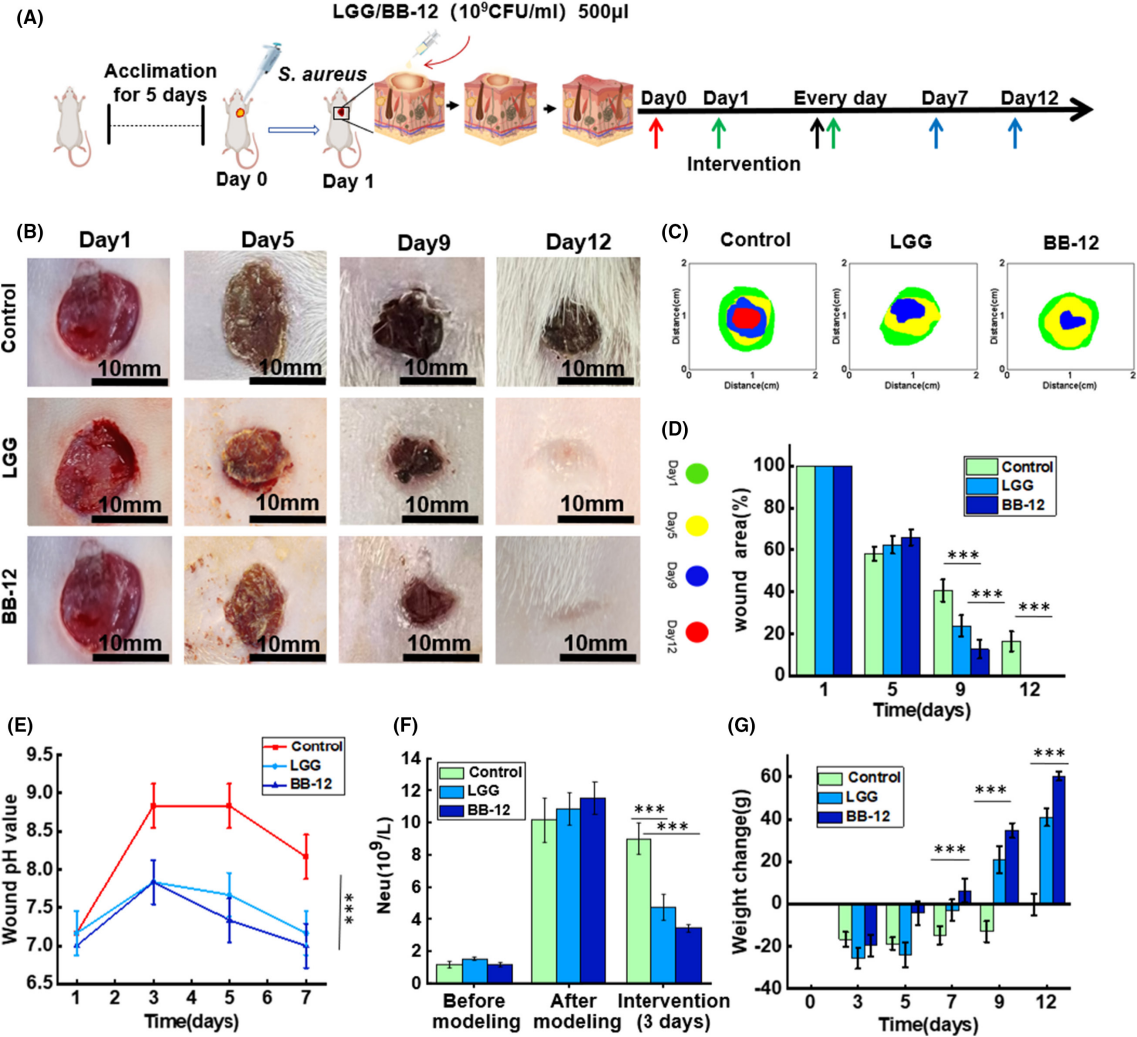
(A) Animal experiment scheme. (B) Photos of wounds at various time points in each group over 12 days. (C) Unhealed wound areas on days 1, 5, 9 and 12. (D) Quantitative analysis of wound area at different time points in each group. (E) Wound pH on days 1, 3, 5 and 7. (F) Neutrophil levels before and after creation of the infected wound model, and 3 days of intervention. (G) Weight changes on days 3, 5, 7, 9 and 12 in each group.

LGG and BB‐12 showed significant inhibitory effects against *S. aureus* (Figure S1 and Table S1), which is one of the most common pathogenic bacteria (Linz et al., 2023). Herein, an *S. aureus*–infected wound rat model was established as described earlier (Han et al., 2024) to evaluate the functions of LGG and BB‐12 in vivo. The modelling process and intervention method are shown in Figure 2A. Topical treatment with LGG and BB‐12 was performed on the wound after successful creation of the model. A representative image of the wound region in each group is shown Figure 2B. Accelerated wound closure was observed in the LGG and BB‐12 groups compared to the control group (Figure 2C). In particular, the wound‐healing rates of the LGG and BB‐12 groups were 76.21% and 87.25%, respectively, after 9 days of intervention, which were significantly higher than that of the control group (59.43%). Notably, the wounds in the LGG and BB‐12 groups completely healed on day 12, whereas the wounds in the control group remained unhealed on day 12 (Figure 2D).

Variations in the wound pH during the healing process are shown in Figure 2E. During the first 3 days, the pH of the wound increased, possibly owing to bacterial infection. From the 4th day, the pH gradually decreased to normal levels. Particularly, on the 3rd day, the pH of the wound in the LGG and BB‐12 groups were 7.83 ± 0.28 and 7.83 ± 0.36, respectively, which were lower than that in the control group (8.83 ± 0.28). Moreover, on the 7th day, the wound pH in the LGG and BB‐12 groups was significantly lower than that in the control group (*p* < 0.001). These results suggest that treatment with LGG and BB‐12 can regulate the microenvironment of infected wounds.

### Wound histological analysis

Haematological analysis revealed that the neutrophil levels in each group increased beyond the normal range (0.35–6.30 × 10^9^/L). After intervention (3 days), the neutrophil level in the LGG and BB‐12 groups was 4.78 ± 0.83 × 10^9^/L and 3.46 ± 0.21 × 10^9^/L respectively. In contrast, neutrophil level in the control group was 9.01 ± 0.98 × 10^9^/L, which is beyond the normal range (Figure 2F). Additionally, weight changes in the rats during the healing process were recorded and are summarized in Figure 2G. Over the first 7 days, the weight of the rats was lower than its initial value owing to infection. After 7 days, it was found that the weight of the rats in LGG and BB‐12 groups increased rapidly, reaching 9.55 ± 7.2 g/day and 10.3 ± 4.1 g/day, which were significantly higher than that of the control group with −0.33 ± 5.32 g/day (*p* < 0.05), suggesting that the infection and inflammation in the rats of the LGG and BB‐12 groups were effectively controlled.

**FIGURE 3:**
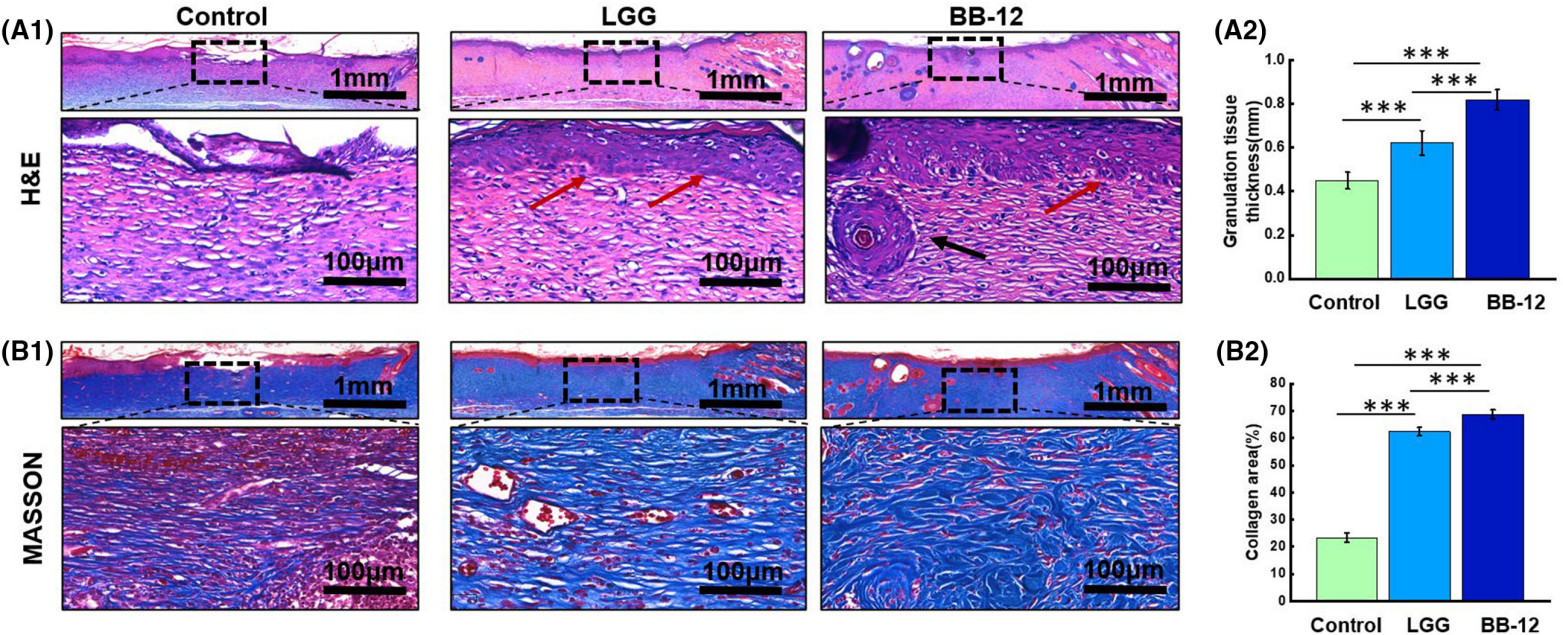
(A_1_) H&E staining images. (A_2_) Granulation tissue thickness. (B_1_) Masson's trichrome staining images. (B_2_) Collagen deposition in the wounds of each group on day 12.

Skin defects are primarily repaired by the in situ growth of granulation tissue (Castaño et al., 2018; Zhong et al., 2022). As shown in Figure 3(A_1_), more granulation was observed in the LGG and BB‐12 groups than in the control group. Furthermore, the granulation tissue in LGG (0.62 ± 0.06 mm) and BB‐12 groups (0.82 ± 0.05 mm) was much thicker than in the control group (0.45 ± 0.04 mm) (Figure 3(A_2_)). Additionally, a higher amount of collagen deposition was observed in LGG (62.4 ± 1.57%) and BB‐12 (68.75 ± 1.63%) groups than in the control group (23.4 ± 1.86%) (Figure 3(B_1_), (B_2_)). Furthermore, the LGG and BB‐12 groups showed almost completely epithelialization (red arrows), accompanied by the appearance of hair follicle structures (black arrows). In contrast, no intact epithelial tissues were observed in the control group.

### Expression of immune cells and inflammatory factors

**FIGURE 4:**
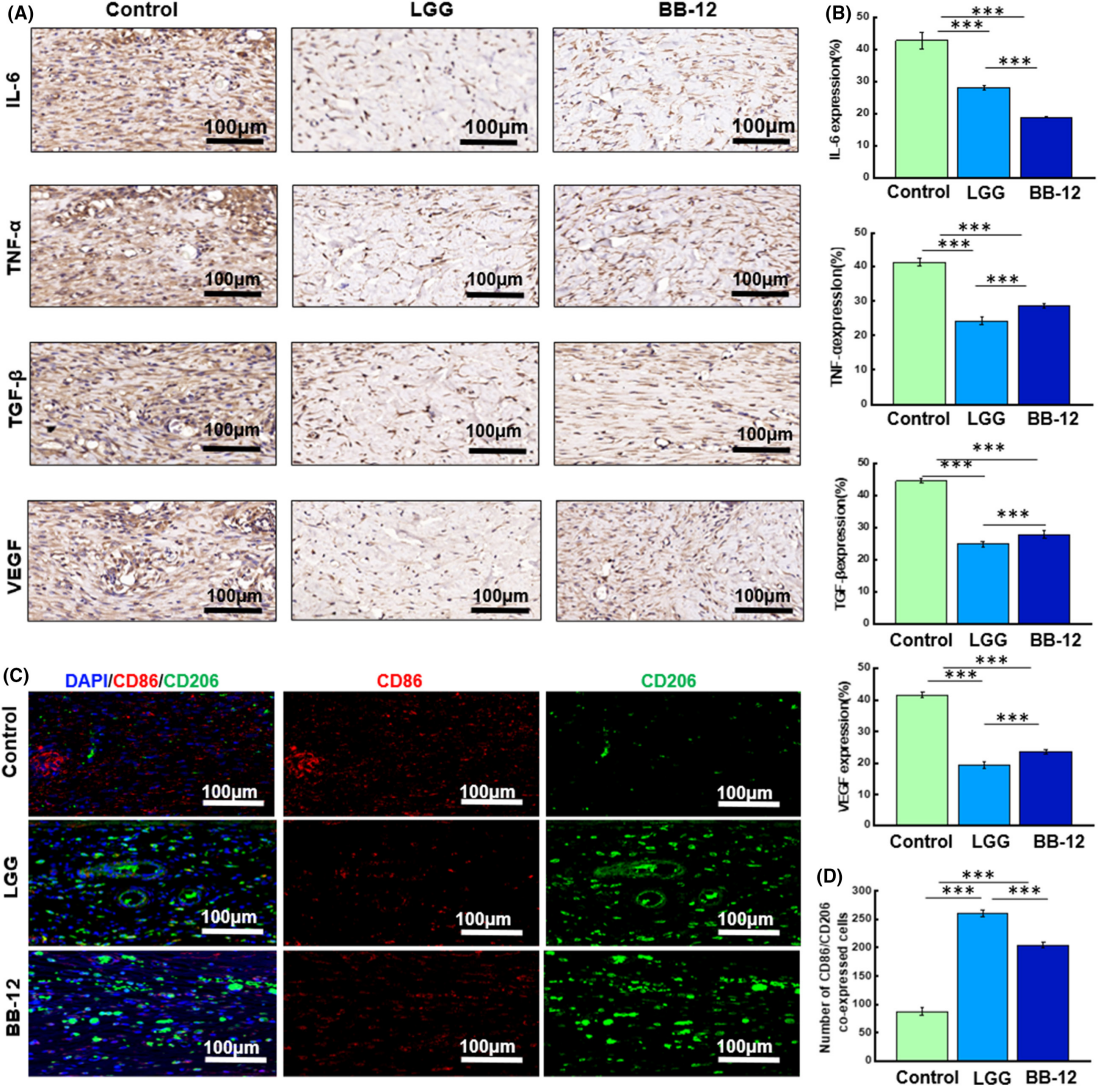
(A) Immunohistochemical staining images and (B) quantification of IL‐6, TNF‐α, TGF‐β and VEGF in skin tissue of infected wounds in each group. (C) Immunofluorescence staining images of M1 macrophage marker CD86 (red) and M2 macrophage marker CD206 (green) in wound skin tissue on day 12 in each group. (D) Quantification of CD86 and CD206 in each group.

TNF‐α and IL‐6 are pro‐inflammatory cytokines that play pivotal roles in the immune response and are often used as markers of inflammation and infection. Herein, the relative levels of TNF‐α and IL‐6 in each group were assessed by immunohistochemical analysis and the results are illustrated in Figure 4A. Compared with the control group, a statistically significant reduction in the expression of IL‐6 and TNF‐α was observed in both LGG and BB‐12 groups (*p* < 0.001). Additionally, growth factors including VEGF and TGF‐β are crucial in the regulation of wound‐healing processes. Immunohistochemistry results and statistical data are shown in Figure 4B, which revealed significantly lower expression levels of TGF‐β in LGG and BB‐12 groups than in the control group on day 12 (*p* < 0.001). Similarly, VEGF expression in LGG and BB‐12 groups was significantly lower than that in the control group (*p* < 0.001). Macrophage distribution and quantity within the wound were assessed using M1 (CD86, red) and M2 (CD206, green) phenotypic markers. Active expression of M1 macrophages can aggravate inflammatory responses. As shown in Figure 4C,D, cells positively stained for CD86 were fewer in LGG and BB‐12 groups than in the control group. Concurrently, a consistent upregulation of CD206‐positive cells was observed in the LGG and BB‐12 groups compared with the control group. These results indicate that topical application of LGG and BB‐12 leads to a shift in macrophages towards a less inflammatory and more reparative phenotype.

### Wound microbial analysis

**FIGURE 5:**
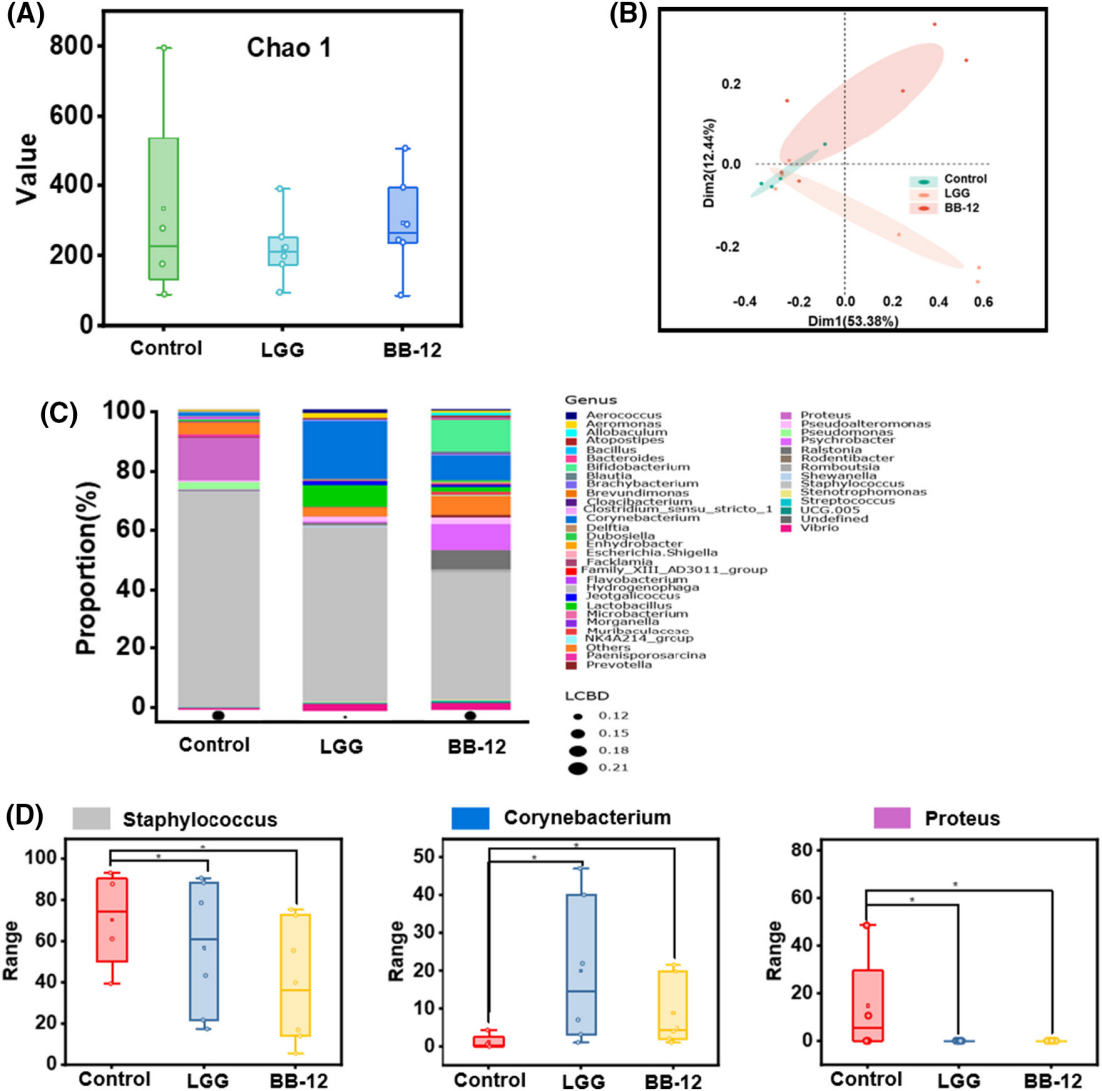
(A) Alpha diversity analysis index Chao1, which is the total number of taxa in the samples and their percentage. (B) The beta diversity. (C) Relative abundance of bacteria from genus level. Relative abundance of (D) *Staphylococcus*, *Corynebacterium* and *Proteus* in each group.

The influence of LGG and BB‐12 interventions on the composition of wound microbiota was investigated. There were no significant changes in the richness and diversity of commensal bacteria among the groups, as evidenced by the Chao indices (Figure 5A). Furthermore, the altered microbial structure was examined using principal coordinate analysis (PCoA) plots at the OUT level, which clearly showed the distance (beta diversity) between the three groups. In addition, the PCoA diagram was divided into three cohesive groups, corresponding to the control, LGG and BB‐12 groups (Figure 5B).

At the genus level, the Wilcoxon rank‐sum test was used to compare the relative abundance of the groups. As illustrated in Figure 5C, the LGG and BB‐12 groups showed significant reductions in the abundance of *Staphylococcus* and *Proteus* compared to that in the control group (*p* < 0.05), along with a notable increase in *Corynebacterium* abundance (*p* < 0.05) (Figure 5D). In particular, *Staphylococcus* displayed the most substantial alteration in abundance, accounting for 70.48%, 58.11% and 40.78% in the control, LGG and BB‐12 groups respectively. These findings suggest that the application of LGG and BB‐12 can effectively modulate the microbial environment in wounds.

## DISCUSSION

This study reveals the significant role of topical probiotics LGG and BB‐12 in regulating the infected wound microenvironment for promotion of wound healing. Particular emphasis has been paid to the ability of LGG and BB‐12 to reshape the wound microbiota, including inhibiting pathogenic bacteria and promoting the proliferation of beneficial microorganisms, thereby enhancing the natural defence mechanism of the wound, which provides new perspectives and solutions in infected wound treatment. Unlike the proprietary probiotics used in previous studies (Chan et al., 2022; Connell et al., 2018), this study selected *LGG* and BB‐12 which are commonly used, safe and readily available strains, and validates their use in actual wound treatment to promote the widespread use of these common strains in clinical scenario.

Consistent with the findings by Moysidis et al. (2022) and Tarapatzi et al. (2022), our study shows that both LGG and BB‐12 significantly enhance the healing of *S. aureus*‐infected wounds. Unlike Panagiotou et al.'s (2023) research, we observed that BB‐12 outperforms LGG in accelerating wound healing. This advantage may be due to the higher concentrations of short‐chain fatty acids (SCFAs), such as acetic acid, propionic acid and butyric acid metabolized by BB‐12, which can inhibit *S. aureus* growth and maintain a proper pH for protease activity. In a study by Twetman et al. (2018), the impact of *Lactobacillus reuteri* on matrix metalloproteinases and interferons in oral mucosa wounds showed no significant differences with the placebo interventions. In contrast, this study revealed that the LGG can promote skin wound healing by regulating the pro‐inflammatory cytokines, TGF‐β, VEGF, macrophage polarization and microbiota. The different results with previous studies suggest that the effects of probiotics should consider the physiological differences of the tissues they are applied to, as well as the distinct bioactivities of the probiotic strains used.

In the early stages of wound healing, granulation tissue lays the foundation for the formation of new blood vessels and cellular matrix. Previous studies have shown that Bifidobacteria can enhance tissue regeneration by remodelling the extracellular matrix and fibroblast migration (O'Connell Motherway et al., 2019; Silva et al., 2018), as well as promote the differentiation and regeneration of epidermal keratinocytes (Szöllősi et al., 2017). This is consistent with our findings that BB‐12 can promote collagen deposition in skin wounds, which may be due to BB‐12 activating key growth factors and signalling pathways, providing a faster and more stable environment for tissue repair. Additionally, several studies (Lam et al., 2007; Mohammedsaeed et al., 2015; Moreira et al., 2021) have shown that *L. rhamnosus* can effectively promote wound healing. The same results have been demonstrated in this study with further evidence that both LGG and BB‐12 can promote collagen deposition.

It is known that probiotic strains have specific effects on immune regulation and tissue healing (Yin et al., 2024). Some studies have shown that lactobacilli can reduce the expression of inflammatory factors (Chen et al., 2024; Zanetta et al., 2023) while other studies found that Bifidobacterium may indirectly affect the host immune system by modulating the gut microbiota, positively impacting wound healing (Almasyan et al., 2023). Another study showed that bifidobacteria can downregulate pro‐inflammatory cytokines thus reducing inflammation and optimizing the healing environment (Feng et al., 2022). Our study, on the one hand, supports these conclusions by demonstrating that both LGG and BB‐12 exhibit significant specificity in modulating immune responses during wound healing, and on the other hand, illustrates the different abilities of the different strains. Specifically, BB‐12 significantly downregulated IL‐6, which is a multifunctional cytokine critical in chronic inflammation and tissue damage (Zhao et al., 2018). Hence, reduction of IL‐6 may help shorten the duration and intensity of inflammation, thereby accelerating wound healing, which is consistent with previous results that inhibition of IL‐6 signalling promotes chronic wound healing (Elhabal et al., 2024). In contrast, LGG significantly reduced TNF‐α, TGF‐β and VEGF. Among them, TNF‐α is a key pro‐inflammatory cytokine secreted by macrophages that initiates and sustains inflammation (Karki et al., 2021). Reduction of TNF‐α may control early inflammation and prevent excessive tissue damage. TGF‐β and VEGF play important roles in different stages of wound healing. TGF‐β promotes fibroblast proliferation and collagen synthesis, accelerating wound closure while excess TGF‐β can lead to scar formation (Wu et al., 2024). VEGF is critical for angiogenesis, providing nutrients and oxygen for wound repair (Wang, Lou, et al., 2022). Downregulation of TGF‐β and VEGF by LGG suggests that it may regulate fibrosis and angiogenesis, promoting healing while preventing tissue overgrowth and abnormal blood vessel formation (Caja et al., 2018; Zhang & Chu, 2019).

Moreover, LGG and BB‐12 exhibit distinct effects on macrophage polarization. BB‐12 significantly reduced the CD86/CD206 ratio, indicating that it can promote the polarization of macrophages from M1 type to M2 type which can secrete anti‐inflammatory cytokines such as IL‐10 (Tu et al., 2021). In contrast, M1 macrophages participate in pro‐inflammatory responses and secrete factors such as TNF‐α to clear pathogens (Qu et al., 2022). Hence, M2 macrophages are critical in the later stages of wound healing (Yang et al., 2024). The primary mechanism of LGG appears to focus on early inflammation, reducing pro‐inflammatory cytokines to prevent further tissue damage (Han et al., 2024). This suggests that the anti‐inflammatory benefits of LGG are valuable for early wound management, whereas BB‐12 may be more effective in promoting tissue repair and regeneration following inflammation.

The modulation of wound microbiota by LGG and BB‐12 found in our study offers new insights into probiotic effects on wound healing. We observed that both LGG and BB‐12 substantially reduced the abundance of pathogenic bacteria, potentially by inhibiting bacterial adhesion and colonization and mitigating harmful metabolites (Chen et al., 2024), thereby improving the wound microenvironment and promoting healing. Notably, BB‐12 demonstrated a superior ability to reduce abundance of *Staphylococcus* which is a common pathogen in chronic wound inflammation (Alabbosh et al., 2023; Liu et al., 2022). Furthermore, there is significant reduction in abundance of *Proteus* spp. which is associated with various infections (Kwiecińska‐Piróg et al., 2020). The marked increase in *Corynebacterium* abundance suggests a potential protective role in the wound (Oh et al., 2016; Sanford & Gallo, 2013). These results imply that both LGG and BB‐12 can not only suppress harmful bacteria but also foster beneficial microbiota to improve the wound microenvironment. The superior performance of BB‐12 in controlling pathogenic bacteria and LGG in promoting beneficial bacteria highlights the specific roles in wound microbiota regulation. This underscores the importance of selecting appropriate probiotics based on microbiota characteristics for wound management. Future studies should further investigate the comprehensive effects of these probiotics on wound healing to optimize clinical application strategies.

## CONCLUSIONS

This study highlights the significant role of topical probiotics of LGG and BB‐12 in modulating the wound microenvironment to promote wound healing. It is evidenced that both LGG and BB‐12 improved wound healing with enhanced granulation tissue formation and collagen deposition. The neutrophil infiltration and the expression of pro‐inflammatory cytokines have been inhibited by both probiotics. Notably, BB‐12 markedly reduced IL‐6, while LGG significantly lowered TNF‐α, TGF‐β and VEGF. Additionally, the probiotics intervention promoted macrophage polarization towards the anti‐inflammatory M2 phenotype. Notably, LGG and BB‐12 significantly decreased the abundance of pathogenic bacteria (e.g. *Staphylococcus* and *Proteus*) and increased the proportion of beneficial bacteria (e.g. *Corynebacterium*). Particularly, BB‐12 was more effective in reducing *Staphylococcus* abundance, whereas LGG excelled in promoting *Corynebacterium* growth. These results provide new theoretical support for probiotic‐based wound treatment strategies for their broader application in clinical practice. Despite the important outcomes, certain limitations exist, such as the reliance on animal models and the lack of coverage for all types of wounds and clinical conditions. Future research should focus on expanding sample sizes, covering various wound models and clinical applications and exploring the specific roles and mechanisms of different probiotic strains in modulating the microbiota. This will help further optimize probiotic therapies, enhance wound care outcomes and offer new solutions for treating complex infections and injuries.

## AUTHOR CONTRIBUTIONS

**Zhe Yin:** Data curation; formal analysis; investigation; methodology; writing – original draft. **Yilin Wang:** Data curation; formal analysis; investigation. **Xiaojuan Feng:** Investigation; methodology. **Changqing Liu:** Methodology. **Shuyan Liu:** Formal analysis; investigation. **Xiaoyang Guan:** Formal analysis. **Zhanyi Long:** Data curation; investigation. **Zhonghua Miao:** Software. **Fang He:** Formal analysis. **Ruyue Cheng:** Methodology. **Yanting Han:** Funding acquisition; investigation; methodology; writing – original draft; writing – review and editing. **Ka Li:** Funding acquisition; project administration; supervision.

## CONFLICT OF INTEREST STATEMENT

The authors declare no conflicts of interest.

## Supporting information


Data S1.

## References

[mbt270031-bib-0001] Alabbosh, K.F. , Zmantar, T. , Bazaid, A.S. , Snoussi, M. & Noumi, E. (2023) Antibiotics resistance and adhesive properties of clinical *Staphylococcus aureus* isolated from wound infections. Microorganisms, 11, 1353. 37317326 10.3390/microorganisms11051353 PMC10221594

[mbt270031-bib-0002] Almasyan, R. , Jafari, P. , Farjanikish, G. & Noorbazargan, H. (2023) Metabiotic extracted from *Bifidobacterium bifidum* modulates antioxidant capacity and inflammatory responses during peptic ulcer healing in male Wistar rats: a preliminary study. Iranian Journal of Microbiology, 15, 102–110. 37069908 10.18502/ijm.v15i1.11924 PMC10105265

[mbt270031-bib-0003] Baquer, F. , Jaulhac, B. , Barthel, C. , Paz, M. , Wolfgramm, J. , Müller, A. et al. (2023) Skin microbiota secretomes modulate cutaneous innate immunity against *Borrelia burgdorferi* s.s. Scientific Reports, 13, 16393. 37773515 10.1038/s41598-023-43566-0 PMC10541882

[mbt270031-bib-0004] Caja, L. , Dituri, F. , Mancarella, S. , Caballero‐Diaz, D. , Moustakas, A. , Giannelli, G. et al. (2018) TGF‐β and the tissue microenvironment: relevance in fibrosis and cancer. International Journal of Molecular Sciences, 19, 1294. 29701666 10.3390/ijms19051294 PMC5983604

[mbt270031-bib-0005] Castaño, O. , Pérez‐Amodio, S. , Navarro‐Requena, C. , Mateos‐Timoneda, M.Á. & Engel, E. (2018) Instructive microenvironments in skin wound healing: biomaterials as signal releasing platforms. Advanced Drug Delivery Reviews, 129, 95–117. 29627369 10.1016/j.addr.2018.03.012

[mbt270031-bib-0006] Chan, P.L. , Lauw, S. , Ma, K.L. , Kei, N. , Ma, K.L. , Wong, Y.O. et al. (2022) ProBioQuest: a database and semantic analysis engine for literature, clinical trials and patents related to probiotics. Database, 2022, baac059. 35849028 10.1093/database/baac059 PMC9290863

[mbt270031-bib-0007] Chen, Y. , Huang, X. , Liu, A. , Fan, S. , Liu, S. , Li, Z. et al. (2024) *Lactobacillus reuteri* vesicles regulate mitochondrial function of macrophages to promote mucosal and cutaneous wound healing. Advanced Science, 11, 2309725. 38647360 10.1002/advs.202309725 PMC11199966

[mbt270031-bib-0008] Cheng, R.Y. , Yao, J.R. , Wan, Q. , Guo, J.W. , Pu, F.F. , Shi, L. et al. (2018) Oral administration of *Bifidobacterium bifidum* TMC3115 to neonatal mice may alleviate IgE‐mediated allergic risk in adulthood. Beneficial Microbes, 9, 815–828. 29888657 10.3920/BM2018.0005

[mbt270031-bib-0009] Chowdhury, F.R. & Findlay, B.L. (2023) Fitness costs of antibiotic resistance impede the evolution of resistance to other antibiotics. ACS Infectious Diseases, 9, 1834–1845. 37726252 10.1021/acsinfecdis.3c00156 PMC10581211

[mbt270031-bib-0010] Connell, M. , Shin, A. , James‐Stevenson, T. , Xu, H. , Imperiale, T.F. & Herron, J. (2018) Systematic review and meta‐analysis: efficacy of patented probiotic, VSL#3, in irritable bowel syndrome. Neurogastroenterology and Motility, 30, e13427. 30069978 10.1111/nmo.13427 PMC6249050

[mbt270031-bib-0011] Constantinides, M.G. , Link, V.M. , Tamoutounour, S. , Wong, A.C. , Perez‐Chaparro, P.J. , Han, S.‐J. et al. (2019) MAIT cells are imprinted by the microbiota in early life and promote tissue repair. Science, 366, eaax6624. 31649166 10.1126/science.aax6624 PMC7603427

[mbt270031-bib-0012] Dhall, S. , Hoffman, T. , Sathyamoorthy, M. , Lerch, A. , Jacob, V. , Moorman, M. et al. (2019) A viable Lyopreserved amniotic membrane modulates diabetic wound microenvironment and accelerates wound closure. Advances in Wound Care, 8, 355–367. 31346490 10.1089/wound.2018.0931 PMC6657363

[mbt270031-bib-0013] Elhabal, S.F. , Abdelaal, N. , Saeed Al‐Zuhairy, S.A.K. , Elrefai, M.F.M. , Elsaid Hamdan, A.M. , Khalifa, M.M. et al. (2024) Green synthesis of zinc oxide nanoparticles from Althaea officinalis flower extract coated with chitosan for potential healing effects on diabetic wounds by inhibiting TNF‐α and IL‐6/IL‐1β signaling pathways. International Journal of Nanomedicine, 19, 3045–3070. 38559447 10.2147/IJN.S455270 PMC10981898

[mbt270031-bib-0014] Enamorado, M. , Kulalert, W. , Han, S.‐J. , Rao, I. , Delaleu, J. , Link, V.M. et al. (2023) Immunity to the microbiota promotes sensory neuron regeneration. Cell, 186, 607–620.e17. 36640762 10.1016/j.cell.2022.12.037 PMC11512587

[mbt270031-bib-0015] Feng, C. , Zhang, W. , Zhang, T. , He, Q. , Kwok, L.‐Y. , Tan, Y. et al. (2022) Heat‐killed *Bifidobacterium bifidum* B1628 may alleviate dextran sulfate sodium‐induced colitis in mice, and the anti‐inflammatory effect is associated with gut microbiota modulation. Nutrients, 14, 5233. 36558391 10.3390/nu14245233 PMC9784753

[mbt270031-bib-0016] Han, Y. , Yin, Z. , Wang, Y. , Jiang, Y. , Chen, J. , Miao, Z. et al. (2024) Photopolymerizable and antibacterial hydrogels loaded with metabolites from *Lacticaseibacillus rhamnosus* GG for infected wound healing. Biomacromolecules, 25, 2587–2596. 38527924 10.1021/acs.biomac.4c00124

[mbt270031-bib-0017] Karki, R. , Sharma, B.R. , Tuladhar, S. , Williams, E.P. , Zalduondo, L. , Samir, P. et al. (2021) Synergism of TNF‐α and IFN‐γ triggers inflammatory cell death, tissue damage, and mortality in SARS‐CoV‐2 infection and cytokine shock syndromes. Cell, 184, 149–168.e17. 33278357 10.1016/j.cell.2020.11.025 PMC7674074

[mbt270031-bib-0018] Knackstedt, R. , Knackstedt, T. & Gatherwright, J. (2020) The role of topical probiotics on wound healing: a review of animal and human studies. International Wound Journal, 17, 1687–1694. 32869480 10.1111/iwj.13451 PMC7949352

[mbt270031-bib-0019] Kwiecińska‐Piróg, J. , Przekwas, J. , Majkut, M. , Skowron, K. & Gospodarek‐Komkowska, E. (2020) Biofilm formation reducing properties of Manuka honey and Propolis in Proteus mirabilis rods isolated from chronic wounds. Microorganisms, 8, 1823. 33228072 10.3390/microorganisms8111823 PMC7699395

[mbt270031-bib-0020] Lam, E.K.Y. , Yu, L. , Wong, H.P.S. , Wu, W.K.K. , Shin, V.Y. , Tai, E.K.K. et al. (2007) Probiotic *Lactobacillus rhamnosus* GG enhances gastric ulcer healing in rats. European Journal of Pharmacology, 565, 171–179. 17395175 10.1016/j.ejphar.2007.02.050

[mbt270031-bib-0021] Linz, M.S. , Mattappallil, A. , Finkel, D. & Parker, D. (2023) Clinical impact of *Staphylococcus aureus* skin and soft tissue infections. Antibiotics, 12, 557. 36978425 10.3390/antibiotics12030557 PMC10044708

[mbt270031-bib-0022] Liu, W. , Gao, R. , Yang, C. , Feng, Z. , Ou‐Yang, W. , Pan, X. et al. (2022) ECM‐mimetic immunomodulatory hydrogel for methicillin‐resistant *Staphylococcus aureus*‐infected chronic skin wound healing. Science Advances, 8, eabn7006. 35857459 10.1126/sciadv.abn7006 PMC9269894

[mbt270031-bib-0023] Lu, Z. , Zhang, H. , Hu, X. , Lu, J. & Wang, D. (2022) Probiotic‐free microfiber membrane for promoting infected wound healing by regulating wound Flora balance. ACS Materials Letters, 4, 2547–2554.

[mbt270031-bib-0024] Mo, R. , Zhang, H. , Xu, Y. , Wu, X. , Wang, S. , Dong, Z. et al. (2023) Transdermal drug delivery via microneedles to mediate wound microenvironment. Advanced Drug Delivery Reviews, 195, 114753. 36828300 10.1016/j.addr.2023.114753

[mbt270031-bib-0025] Mohammedsaeed, W. , Cruickshank, S. , McBain, A.J. & O'Neill, C.A. (2015) *Lactobacillus rhamnosus* GG lysate increases re‐epithelialization of keratinocyte scratch assays by promoting migration. Scientific Reports, 5, 16147. 26537246 10.1038/srep16147 PMC4633615

[mbt270031-bib-0026] Moreira, C.F. , Cassini‐Vieira, P. , Canesso, M.C.C. , Felipetto, M. , Ranfley, H. , Teixeira, M.M. et al. (2021) Lactobacillus rhamnosus CGMCC 1.3724 (LPR) improves skin wound healing and reduces scar formation in mice. Probiotics and Antimicrobial Proteins, 13, 709–719. 33433898 10.1007/s12602-020-09713-z

[mbt270031-bib-0027] Moysidis, M. , Stavrou, G. , Cheva, A. , Abba Deka, I. , Tsetis, J.K. , Birba, V. et al. (2022) The 3‐D configuration of excisional skin wound healing after topical probiotic application. Injury, 53, 1385–1393. 35148901 10.1016/j.injury.2022.02.006

[mbt270031-bib-0028] O'Connell Motherway, M. , Houston, A. , O'Callaghan, G. , Reunanen, J. , O'Brien, F. , O'Driscoll, T. et al. (2019) A bifidobacterial pilus‐associated protein promotes colonic epithelial proliferation. Molecular Microbiology, 111, 287–301. 30352131 10.1111/mmi.14155

[mbt270031-bib-0029] Oh, J. , Byrd, A.L. , Park, M. , NISC Comparative Sequencing Program , Kong, H.H. & Segre, J.A. (2016) Temporal stability of the human skin microbiome. Cell, 165, 854–866. 27153496 10.1016/j.cell.2016.04.008 PMC4860256

[mbt270031-bib-0030] Ong, J.S. , Taylor, T.D. , Yong, C.C. , Khoo, B.Y. , Sasidharan, S. , Choi, S.B. et al. (2020) *Lactobacillus plantarum* USM8613 aids in wound healing and suppresses *Staphylococcus aureus* infection at wound sites. Probiotics and Antimicrobial Proteins, 12, 125–137. 30659503 10.1007/s12602-018-9505-9

[mbt270031-bib-0031] Panagiotou, D. , Filidou, E. , Gaitanidou, M. , Tarapatzi, G. , Spathakis, M. , Kandilogiannakis, L. et al. (2023) Role of *Lactiplantibacillus plantarum* UBLP‐40, *Lactobacillus rhamnosus* UBLR‐58 and *Bifidobacterium longum* UBBL‐64 in the wound healing process of the excisional skin. Nutrients, 15, 1822. 37111041 10.3390/nu15081822 PMC10141733

[mbt270031-bib-0032] Partlow, J. , Blikslager, A. , Matthews, C. , Law, M. , Daniels, J. , Baker, R. et al. (2016) Effect of topically applied *Saccharomyces boulardii* on the healing of acute porcine wounds: a preliminary study. BMC Research Notes, 9, 210. 27067538 10.1186/s13104-016-2012-8 PMC4827247

[mbt270031-bib-0033] Qu, M. , Zhu, H. & Zhang, X. (2022) Extracellular vesicle‐mediated regulation of macrophage polarization in bacterial infections. Frontiers in Microbiology, 13, 1039040. 36619996 10.3389/fmicb.2022.1039040 PMC9815515

[mbt270031-bib-0034] Reigadas, E. , van Prehn, J. , Falcone, M. , Fitzpatrick, F. , Vehreschild, M.J.G.T. , Kuijper, E.J. et al. (2021) How to: prophylactic interventions for prevention of *Clostridioides difficile* infection. Clinical Microbiology and Infection, 27, 1777–1783. 34245901 10.1016/j.cmi.2021.06.037

[mbt270031-bib-0035] Ren, D. , Gong, S. , Shu, J. , Zhu, J. , Rong, F. , Zhang, Z. et al. (2017) Mixed *Lactobacillus plantarum* strains inhibit *Staphylococcus aureus* induced inflammation and ameliorate intestinal microflora in mice. BioMed Research International, 2017, e7476467. 10.1155/2017/7476467 PMC5551470 28819629

[mbt270031-bib-0036] Salisbury, A.‐M. , Woo, K. , Sarkar, S. , Schultz, G. , Malone, M. , Mayer, D.O. et al. (2018) Tolerance of biofilms to antimicrobials and significance to antibiotic resistance in wounds. Surgical Technology International, 33, 59–66. 30326137

[mbt270031-bib-0037] Sanford, J.A. & Gallo, R.L. (2013) Functions of the skin microbiota in health and disease. Seminars in Immunology, 25, 370–377. 24268438 10.1016/j.smim.2013.09.005 PMC4219649

[mbt270031-bib-0038] Scharschmidt, T.C. , Vasquez, K.S. , Pauli, M.L. , Leitner, E.G. , Chu, K. , Truong, H.‐A. et al. (2017) Commensal microbes and hair follicle morphogenesis coordinately drive Treg migration into neonatal skin. Cell Host & Microbe, 21, 467–477.e5. 28343820 10.1016/j.chom.2017.03.001 PMC5516645

[mbt270031-bib-0039] Silva, A.K.S. , Silva, T.R.N. , Nicoli, J.R. , Vasquez‐Pinto, L.M.C. & Martins, F.S. (2018) In vitro evaluation of antagonism, modulation of cytokines and extracellular matrix proteins by Bifidobacterium strains. Letters in Applied Microbiology, 67, 497–505. 30099746 10.1111/lam.13062

[mbt270031-bib-0040] Szöllősi, A.G. , Gueniche, A. , Jammayrac, O. , Szabó‐Papp, J. , Blanchard, C. , Vasas, N. et al. (2017) *Bifidobacterium longum* extract exerts pro‐differentiating effects on human epidermal keratinocytes, in vitro. Experimental Dermatology, 26, 92–94. 27315170 10.1111/exd.13130

[mbt270031-bib-0041] Tarapatzi, G. , Filidou, E. , Kandilogiannakis, L. , Spathakis, M. , Gaitanidou, M. , Arvanitidis, K. et al. (2022) The probiotic strains *Bifidοbacterium lactis*, *Lactobacillus acidophilus*, *Lactiplantibacillus plantarum* and *Saccharomyces boulardii* regulate wound healing and chemokine responses in human intestinal subepithelial myofibroblasts. Pharmaceuticals (Basel), 15, 1293. 36297405 10.3390/ph15101293 PMC9611312

[mbt270031-bib-0042] Tu, Z. , Chen, M. , Wang, M. , Shao, Z. , Jiang, X. , Wang, K. et al. (2021) Engineering bioactive M2 macrophage‐polarized anti‐inflammatory, antioxidant, and antibacterial scaffolds for rapid angiogenesis and diabetic wound repair. Advanced Functional Materials, 31, 2100924.

[mbt270031-bib-0043] Twetman, S. , Pedersen, A.M.L. & Yucel‐Lindberg, T. (2018) Probiotic supplements containing *Lactobacillus reuteri* does not affect the levels of matrix metalloproteinases and interferons in oral wound healing. BMC Research Notes, 11, 759. 30359300 10.1186/s13104-018-3873-9 PMC6203191

[mbt270031-bib-0044] Wang, C.‐G. , Lou, Y.‐T. , Tong, M.‐J. , Zhang, L.‐L. , Zhang, Z.‐J. , Feng, Y.‐Z. et al. (2022) Author correction: Asperosaponin VI promotes angiogenesis and accelerates wound healing in rats via up‐regulating HIF‐1α/VEGF signaling. Acta Pharmacologica Sinica, 43, 1617–1618. 34526673 10.1038/s41401-021-00777-3 PMC9160048

[mbt270031-bib-0045] Wang, G. , Sweren, E. , Liu, H. , Wier, E. , Alphonse, M.P. , Chen, R. et al. (2021) Bacteria induce skin regeneration via IL‐1β signaling. Cell Host & Microbe, 29, 777–791.e6. 33798492 10.1016/j.chom.2021.03.003 PMC8122070

[mbt270031-bib-0046] Wang, Z. , Qi, F. , Luo, H. , Xu, G. & Wang, D. (2022) Inflammatory microenvironment of skin wounds. Frontiers in Immunology, 13, 789274. 35300324 10.3389/fimmu.2022.789274 PMC8920979

[mbt270031-bib-0047] Wu, J. , Song, Y. , Wang, J. , Wang, T. , Yang, L. , Shi, Y. et al. (2024) Isorhamnetin inhibits hypertrophic scar formation through TGF‐β1/Smad and TGF‐β1/CREB3L1 signaling pathways. Heliyon, 10, e33802. 39055792 10.1016/j.heliyon.2024.e33802 PMC11269880

[mbt270031-bib-0048] Yang, C. , Ma, X. , Wu, P. , Shang, L. , Zhao, Y. & Zhong, L. (2023) Adhesive composite microspheres with dual antibacterial strategies for infected wound healing. Small, 19, 2301092. 10.1002/smll.202301092 37069775

[mbt270031-bib-0049] Yang, Y. , Fan, L. , Jiang, J. , Sun, J. , Xue, L. , Ma, X. et al. (2024) M2 macrophage‐polarized anti‐inflammatory microneedle patch for accelerating biofilm‐infected diabetic wound healing via modulating the insulin pathway. Journal of Nanobiotechnology, 22, 489. 39143532 10.1186/s12951-024-02731-x PMC11323363

[mbt270031-bib-0050] Yin, Z. , Qiu, Y. , Han, Y. & Li, K. (2024) Topical probiotics in wound care: a review of effects, mechanisms, and applications. Interdisciplinary Nursing Research, 3, 63.

[mbt270031-bib-0051] Zanetta, P. , Ballacchino, C. , Squarzanti, D.F. , Amoruso, A. , Pane, M. & Azzimonti, B. (2023) *Lactobacillus johnsonii* LJO02 (DSM 33828) cell‐free supernatant and vitamin D improve wound healing and reduce Interleukin‐6 production in *Staphylococcus aureus*‐infected human keratinocytes. Pharmaceutics, 16, 18. 38276496 10.3390/pharmaceutics16010018 PMC10820395

[mbt270031-bib-0052] Zhang, J. & Chu, M. (2019) Differential roles of VEGF: relevance to tissue fibrosis. Journal of Cellular Biochemistry, 120, 10945–10951. 30793361 10.1002/jcb.28489

[mbt270031-bib-0053] Zhang, X. , Feng, J. , Feng, W. , Xu, B. , Zhang, K. , Ma, G. et al. (2022) Glycosaminoglycan‐based hydrogel delivery system regulates the wound microenvironment to rescue chronic wound healing. ACS Applied Materials & Interfaces, 14, 31737–31750. 35802505 10.1021/acsami.2c08593

[mbt270031-bib-0054] Zhao, S. , Adebiyi, M.G. , Zhang, Y. , Couturier, J.P. , Fan, X. , Zhang, H. et al. (2018) Sphingosine‐1‐phosphate receptor 1 mediates elevated IL‐6 signaling to promote chronic inflammation and multitissue damage in sickle cell disease. The FASEB Journal, 32, 2855–2865. 29401601 10.1096/fj.201600788RR PMC5901384

[mbt270031-bib-0055] Zhong, J. , Wang, H. , Yang, K. , Wang, H. , Duan, C. , Ni, N. et al. (2022) Reversibly immortalized keratinocytes (iKera) facilitate re‐epithelization and skin wound healing: potential applications in cell‐based skin tissue engineering. Bioactive Materials, 9, 523–540. 34820586 10.1016/j.bioactmat.2021.07.022 PMC8581279

