# Correction to ‘*Lactobacillus rhamnosus* GG and *Bifidobacterium animalis* subsp. *lactis* BB‐12 Promote Infected Wound Healing via Regulation of the Wound Microenvironment’

**DOI:** 10.1111/1751-7915.70486

**Published:** 2026-11-10

Yin, Z. , Y. Wang , X. Feng , et al. 2024. “
*Lactobacillus rhamnosus* GG and *Bifidobacterium animalis* subsp. *lactis* BB‐12 Promote Infected Wound Healing via Regulation of the Wound Microenvironment.” Microbial Biotechnology 17: e70031. 10.1111/1751-7915.70031.PMC1148811839422648

Following a careful post‐publication review of our original experimental datasets, we identified an error in Figure 2B. Specifically, the representative wound image for the Control group at Day 1 was inadvertently duplicated with the corresponding image from the BB‐12 group. We have re‐examined all original experimental records and image archives, and the correct representative image for the Control group at Day 1 has been identified and verified. Accordingly, Figure 2 has been revised to ensure the accurate presentation of all experimental groups. We confirm that this correction does not affect the quantitative analyses, results, interpretation, or conclusions of the study.

The correct figure 2 is provided below.

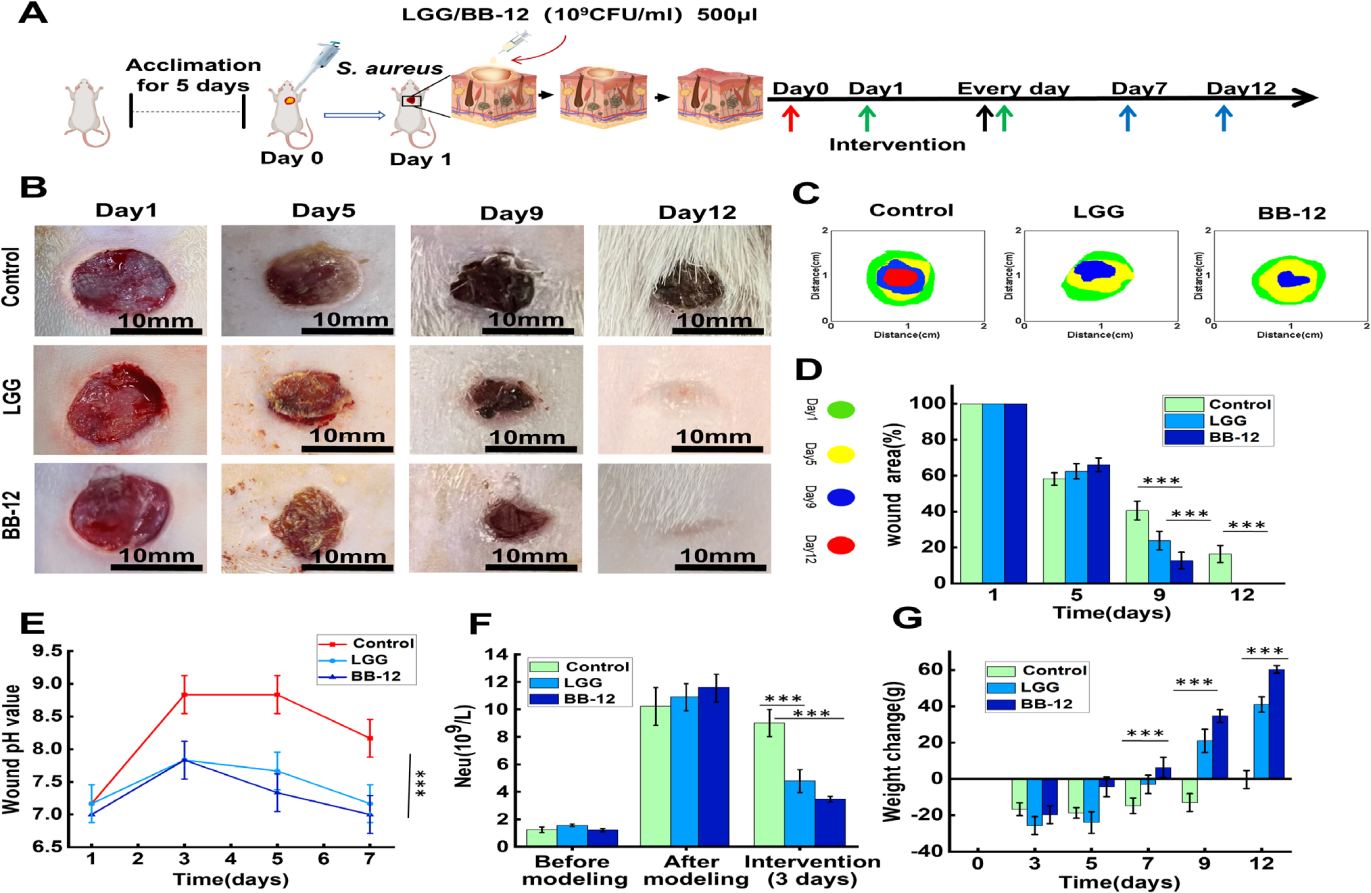

We apologize for this error.

