# Supplementary material for: Lactobacillus rhamnosus GG and Bifidobacterium animalis subsp. lactis BB‐12 promote infected wound healing via regulation of the wound microenvironment
Source: Microb Biotechnol. 2024 Oct 18;17(10):e70031. doi: 10.1111/1751-7915.70031 (PMC11488118; doi:10.1111/1751-7915.70031)
Supplement: Supplementary file 1 — Data S1. [file MBT2-17-e70031-s001.docx]

***Lactobacillus rhamnosus* GG and** ***Bifidobacterium animalis* subsp*. lactis* BB-12 promote infected wound healing via regulation of the wound microenvironment**

YIN Zhe ^a,b,c^, WANG Yilin ^b^, FENG Xiaojuan ^d^, Changqing Liu ^b^, Xiaoyang Guan ^e^,

LIU Shuyan ^b^, LONG Zhanyi ^b^, MIAO Zhonghua ^f^, HE Fang ^g^, CHENG Ruyue ^g^*, HAN Yanting ^b^*, LI Ka ^b^*

^a^ Sichuan University-The Hong Kong Polytechnic University Institute for Disaster Management and Reconstruction, Chengdu, China

^b^ Medicine and Engineering Interdisciplinary Research Laboratory of Nursing & Materials, West China Hospital, Sichuan University/West China School of Nursing, Sichuan University, Chengdu 610041, China

^c^ Department of Gastroenterology, Affiliated Tumor Hospital of Xinjiang Medical University, Urumqi, China

^d^ Department of General Surgery, West China Hospital, Sichuan University, Chengdu, China

^e^ School of Fashion and Textiles, The Hong Kong Polytechnic University, Hung Hom, Kowloon, Hong Kong SAR, 999077 China

^f^ Department of Clinical Nutrition, West China Second Hospital, Sichuan University, Chengdu, China

^g^ Department of Nutrition and Food Hygiene, West China School of Public Health and West China Fourth Hospital, Sichuan University, Chengdu, China

*Corresponding author: Dr CHENG Ruyue (E-mail: [ruyuecheng1993@163.com](mailto:ruyuecheng1993@163.com)), Dr HAN Yanting (E-mail: [yanthan@126.com](mailto:yanthan@126.com)), and Prof LI Ka (E-mail: [lika127@126.com](mailto:lika127@126.com))

**1. *In vitro* antibacterial experiments**

**1.1 Antibacterial activity measured via inhibition zones on agar**

The in vitro antibacterial properties of LGG and BB-12 were tested at 10^8^ CFU/mL and 10^9^ CFU/mL. First, 100 μL of *S. aureus* (10 ^6^ CFU/mL) and *E. coli* (10^7^ CFU/mL) cultures were spread evenly on the surface of the agar plate. Then, three Oxford cups were placed at equal distances and 200 μL of the probiotic components (10^8^ CFU/mL and 10^9^ CFU/mL) to was added each Oxford cup. After incubation at 37 °C for 24 hours, the diameter of the inhibition zone was measured using a vernier caliper.

**1.2 Antibacterial activity in co-culture**

*S. aureus* and *E. coli* were co-cultured with each of the probiotic components (LGG and BB-12) for 24 hours. After this, the cultures were spread on an agar plate and incubated at 37℃ for 24 hours and the number of *S. aureus* or *E. coli* colonies was counted. The antibacterial rate was calculated according to the following equation:

Antibacterial rate = (Nc - Nt) / Nc × 100%

Where Nc and Nt are the number of colonies in the control (without LGG and BB-12) and treatment groups, respectively.

**2 Results**

**2.1 *In vitro* antibacterial properties**

Oxford cup assays were performed to evaluate the antibacterial activities of LGG and BB-12 at two concentrations (10^8^ and 10^9^ CFU/mL) against *S. aureus* and *E. coli*. The inhibition zones were measured as shown in **Figure S1(A)**. It was evident that LGG and BB-12, at the higher concentration of 10^9^ CFU/mL, had stronger inhibitory activity against *S. aureus* and *E. coli*. Notably, both LGG and BB-12 demonstrated superior inhibitory activity against *S. aureus* than against *E. coli*. The antibacterial effect of LGG on *E. coli* surpassed that of BB-12 (*p*<0.001), as illustrated in **Figure S1(B)** and **Figure S1(C)**. The antibacterial performance was further verified using plate count results. The numbers of *S. aureus* and *E. coli* colonies in the LGG and BB-12 groups were significantly lower than those in the control group (**Figure S**1D). The antibacterial activity of LGG against *S. aureus* and *E. coli* was 100%. For BB-12, the antibacterial rates against *S. aureus* were 96.80±0.03% whereas the antibacterial rates against *E. coli* were 92.00±0.05% (**Table 1**).


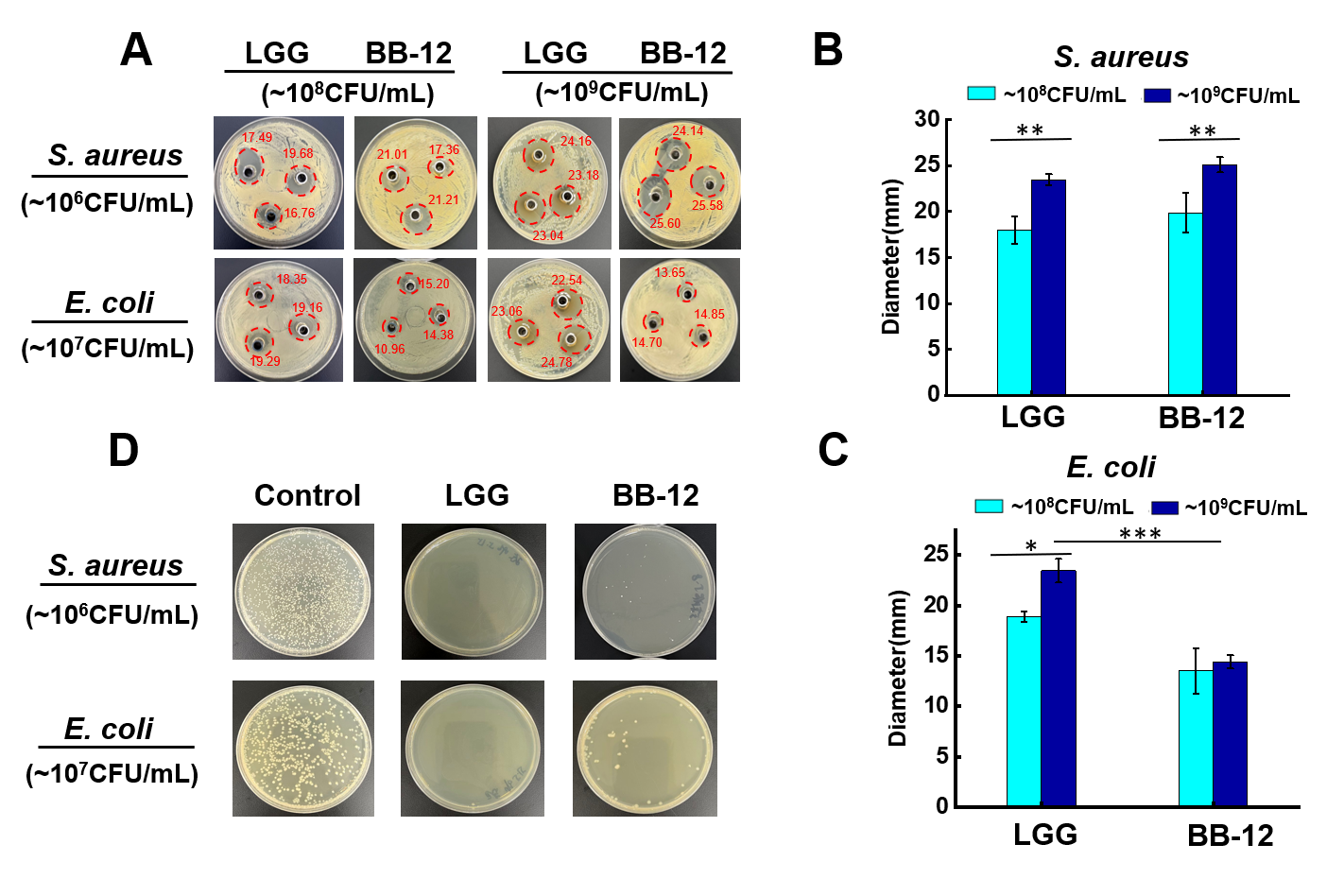


**Figure S1** Oxford cup experiment results of LGG and BB-12 at concentrations of (**A)**10^8^ and 10^9^ CFU/mL. The inhibitory diameter of LGG and BB-12, (10^8^ CFU/mL and 10^9^ CFU/mL) against (**B)** *S. aureus* and (**C**) *E. coli*. (**D)** Plate counting experimental results of LGG and BB-12 (10^9^CFU/mL) in which the concentration of *S. aureus* and *E. coli* was 10^6^ CFU/mL and 10^7^ CFU/mL, respectively.

**Table 1**  The antibacterial activity of LGG and BB-12

| Strains | Group | The viable colonies (CFU/mL) | Killing rate（%） |
| --- | --- | --- | --- |
| *S. aureus* | Control | 4.69×10^6^ | 0 |
|  | LGG | 0 | 100±0 |
|  | BB-12 | 0.15×10^6^ | 96.80±0.03 |
| *E. coli* | Control | 5.88×10^6^ | 0 |
|  | LGG | 0 | 100±0 |
|  | BB-12 | 0.47×10^6^ | 92.00±0.05 |

**Reference**

1. Leser, T., & Baker, A. (2024). Molecular Mechanisms of *Lacticaseibacillus rhamnosus*, LGG® Probiotic Function. Microorganisms, 12(4), 794.
2. Van Holm, W., Carvalho, R., Delanghe, L., Eilers, T., Zayed, N., Mermans, F., ... & Teughels, W. (2023). Antimicrobial potential of known and novel probiotics on in vitro periodontitis biofilms. NPJ biofilms and microbiomes, 9(1), 3.
3. Roozbahani, F., Ahanjan, M., Moshiri, M., Abediankenari, S., Goli, H. R., Kakavan, M., & Gholami, M. (2024). Characterization of Antimicrobial Activities of *Bifidobacterium lactis* BB-12 and Their Inhibitory Effect Against Some Foodborne Pathogens. Foodborne Pathogens and Disease.
